# Interval mapping for red/green skin color in Asian pears using a modified QTL-seq method

**DOI:** 10.1038/hortres.2017.53

**Published:** 2017-10-04

**Authors:** Huabai Xue, Ting Shi, Fangfang Wang, Huangkai Zhou, Jian Yang, Long Wang, Suke Wang, Yanli Su, Zhen Zhang, Yushan Qiao, Xiugen Li

**Affiliations:** 1 College of Horticulture, Nanjing Agricultural University, Nanjing 210095, China; 2 Zhengzhou Fruit Research Institute, Chinese Academy of Agricultural Sciences (CAAS), Key Laboratory of Fruit Breeding Technology of Ministry of Agriculture, Zhengzhou 450009, China; 3 Guangzhou Gene Denovo Biotechnology Co. Ltd, Guangzhou 510006, China

## Abstract

Pears with red skin are attractive to consumers and provide additional health benefits. Identification of the gene(s) responsible for skin coloration can benefit cultivar selection and breeding. The use of QTL-seq, a bulked segregant analysis method, can be problematic when heterozygous parents are involved. The present study modified the QTL-seq method by introducing a |Δ(SNP-index)| parameter to improve the accuracy of mapping the red skin trait in a group of highly heterozygous Asian pears. The analyses were based on mixed DNA pools composed of 28 red-skinned and 27 green-skinned pear lines derived from a cross between the ‘Mantianhong’ and ‘Hongxiangsu’ red-skinned cultivars. The ‘Dangshansuli’ cultivar genome was used as reference for sequence alignment. An average single-nucleotide polymorphism (SNP) index was calculated using a sliding window approach (200-kb windows, 20-kb increments). Nine scaffolds within the candidate QTL interval were in the fifth linkage group from 111.9 to 177.1 cM. There was a significant linkage between the insertions/deletions and simple sequence repeat markers designed from the candidate intervals and the red/green skin (R/G) locus, which was in a 582.5-kb candidate interval that contained 81 predicted protein-coding gene models and was composed of two subintervals at the bottom of the fifth chromosome. The ZFRI 130-16, In2130-12 and In2130-16 markers located near the R/G locus could potentially be used to identify the red skin trait in Asian pear populations. This study provides new insights into the genetics controlling the red skin phenotype in this fruit.

## Introduction

Pear, *Pyrus* L. (tribe Maleae, subfamily Amygdaloideae, family Rosaceae), is an important temperate fruit crop.^1^ In China (2014), the area used for pear production exceeded 1.12 million ha with a harvest of 18.10 million tons, accounting for 70.1% of global pear production (FAOSTAT, http://faostat.fao.org/). Pear fruit color is a major characteristic feature and is an important marketable trait.^2^ Green-, yellow- and russet brown-skinned pear cultivars are common in China, but red-skinned cultivars are rare.^3–5^ Previous research has indicated that consumers have a strong preference for red-skinned pears.^6^ In addition to the aesthetic appeal of their color, the anthocyanins in the red skin of pears have been shown to provide antioxidant benefits for human health.^7,8^ Compared with European pear cultivars, there are few full red-skinned Asian pear cultivars,^4,9^ and the red color usually appears on the side of the fruit that is exposed to the sun in these cultivars.

Although there is definite genetic governance of the trait, the extent of the red skin color of pear fruit is largely dependent on environmental conditions^10^ and is primarily because of the presence of anthocyanins.^11–13^ The biosynthesis and accumulation of anthocyanins are mainly determined by genetic factors and are influenced by light, temperature and other environmental factors.^14^ The peak of anthocyanin biosynthesis occurs in the middle stage of fruit development in *P. communis* L. and some Asian pear cultivars^15–17^ and at harvest in other Asian pear cultivars, a trend similar to that in apples.^18,19^ In pears, most of the structural and regulatory genes in the anthocyanin biosynthetic pathway have been identified and functionally characterized.^20–24^ Phenylalanine ammonia-lyase (PAL) and UDP-glucose: flavonoid-3-O-glucosyltransferase (UFGT) participate in the first and final steps of anthocyanin biosynthesis, respectively. The correlation between anthocyanin biosynthesis and PAL and UFGT activity is obvious in blushed pears but not in full red European pear fruits.^19,25^ In *P. pyrifolia* red-skinned ‘Mantianhong’ and ‘Aoguan’ cultivars, chalcone isomerase (CHI) activity, not PAL, was found to be closely related to anthocyanin biosynthesis and accumulation.^17^ A study on two other red-skinned pear cultivars, ‘Meirensu’ and ‘Yunhongli No. 1’, revealed that PAL induced anthocyanin biosynthesis and that UFGT was closely related to anthocyanin accumulation.^18^ Research has demonstrated that the varying levels of expression of anthocyanidin synthase (*ANS*), *UFGT* and tryptophan-aspartic acid repeat protein (*WD40*) genes and the coexpression of *MYB10* and *bHLH33* (basic helix–loop–helix 33) genes in anthocyanin biosynthesis form the basis for the differences in coloration between Asian and European pears.^9,12^ Additionally, the differential impact of the MYB–bHLH–WD40 complex on the expression of pear anthocyanin structural genes led to differences in the color pattern of red-skinned pears. A correlation between hypermethylation and the green-skinned pear phenotype was found by comparing the methylation levels in the *PcMYB10* promoter in ‘Max Red Bartlett’ (*P. communis* L.) and its green-skinned mutant.^25^

The full red-skinned phenotype of the European pear ‘Max Red Bartlett’, a red bud mutant of ‘Bartlett’, is controlled by a dominant gene *C*.^26^ Pierantoni *et al.*^24^ mapped the position of *PcMYB10* (LG9), noting that it corresponded to the same location as *MdMYBa* and *MdMYB10* and that it controls the pigmentation of apple skin. *PcMYB10*, however, was not directly responsible for the red or yellow skin color in the above pear cultivars because the mutation underlying this color difference maps to a different region of the pear genome. Similarly, in the F1 hybrids of ‘BonRouge’ and ‘Packham's Triumph’ cultivars, the ratio of red- to green-skinned fruits was 1:1, which obeys Mendel's laws of inheritance.^27^ In addition, Dondini *et al*.^13^ analyzed seven crosses from red- and green-skinned European pears, reporting that the red-skinned phenotype in full-colored pears was controlled by a single dominant gene and that the dominant gene *Red* was located on linkage group four (LG4) of ‘Max Red Bartlett’. In contrast, researchers studied the F1 hybrids of two crosses from ‘Huobali’, a red-skinned Asian pear cultivar that is considered to have the genetic predisposition for russet brown, yellow and green skin coloration to be dominant over red skin coloration.^28^ Volz *et al*.^29^ analyzed red skin color segregation ratios in 10 Asian pear hybrid families derived from ‘Huobali’ and suggested that genetic control of blush color in this cultivar may be due to the complimentary action of at least two dominant genes. These results indicate that the genetic basis and mechanism for red skin color may differ in Asian and European pear cultivars.

Michelmore *et al*.^30^ developed a bulked segregant analysis (BSA) method for rapidly identifying markers linked to any specific gene or genomic region and used this method to identify three randomly amplified polymorphic DNA markers linked to downy mildew resistance in lettuce. The BSA method is applicable to qualitative trait gene and quantitative trait loci (QTL) analyses.^31^ With the development and widespread use of high-throughput whole-genome sequencing technology, BSA analysis has become a fast, efficient and widely used method for identifying gene mutations and their locations in the genome.^32^ Advances in the use of X-QTL,^33,34^ MutMap,^35^ MMAPPR^32^ and QTL-seq^36^ have been recently reported. Takagi *et al*.^36^ identified QTLs for partial resistance to rice blast disease and seedling vigor using QTL-seq on recombinant inbred lines and F2 populations of rice. They used whole-genome resequencing of two DNA bulks of progeny (20–50 individuals each) that exhibited extremely contrasting phenotypes. The DNA bulks were aligned to the reference sequence of one parental line to calculate the single-nucleotide polymorphism-index (SNP-index), and QTLs were identified as peaks or valleys in the SNP-index plot. This method can be generally applied in population genomic studies to rapidly identify genomic regions associated with specific traits. QTL-seq has been successfully applied in rice,^36,37^ foxtail millet,^38^ cucumber,^39^ tomato,^40^ chickpea^41–43^ and peach.^44^

Little information has been reported on the hereditary law of the red color trait in Asian pears. Because there is no significant correlation between the red-skinned trait and the color of young shoots and leaves, it has not been possible to make an initial selection for red-skinned fruit at the seedling stage by examining shoot or leaf color. Unfortunately, there are no molecular markers for the red- or green-skinned phenotypes. Therefore, the present study used a modified QTL-seq method to map the red or green skin trait in a segregating population of two red-skinned Asian pears. The initial focus of the study was to identify molecular markers that could be used in a marker-assisted breeding program. This study also provides a solid foundation for identifying the gene responsible for red-skinned fruit and the mechanisms involved in the development of red skin in Asian pears.

## Materials and methods

### Plant material

**Figure 1:**
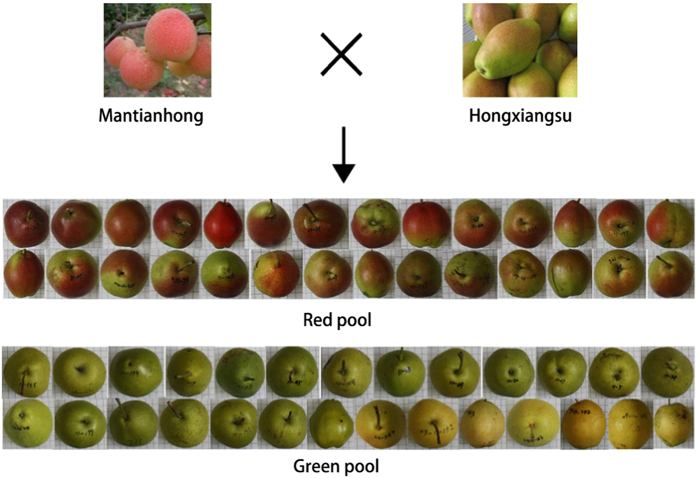
Fruit skin color phenotype of ‘Mantianhong’, ‘Hongxiangsu’ and the extreme phenotypes in the pooled red and green skinned individuals. Note: Both the female parent ‘Mantianhong’ and the male parent ‘Hongxiangsu’ of the population are with red skinned phenotypes. The majority of fruits of plants selected for the red pool exhibited a red blush over 50% of their fruit surface. All fruits of plants selected to represent the extreme phenotype of the green pool were obviously green to yellow and with no red blush apparent on their fruit surface.

The red-skinned Asian pear cultivars ‘Mantianhong’ (*P. pyrifolia*), ‘Hongxiangsu’ (*P. pyrifolia* White Pear Group) and ‘Yuluxiang’ (*P. pyrifolia* White Pear Group) were grown as the parental lines in the orchard of the Zhengzhou Fruit Research Institute (ZFRI), Chinese Academy of Agricultural Sciences (CAAS) in Zhengzhou (Henan Province, China). One F1 hybrid population resulting from a cross between ‘Mantianhong’ and ‘Hongxiangsu’ (*n*=348) and another F1 hybrid population resulting from a cross between ‘Yuluxiang’ and ‘Mantianhong’ (*n*=458) were used as the study material. The populations were established in 2009 and grown at the affiliated experimental orchard of the ZFRI, CAAS in Xinxiang (Henan Province, China). Most individuals bore their first fruit in 2013. Trees bearing one or more fruits with a red blush surface (>0%) were considered the red-skinned phenotype, and trees with green to yellow coloration on all fruit surfaces were considered the green-skinned phenotype. Individuals with extreme red or green skin coloration in the population, resulting from the cross between the ‘Mantianhong’ and ‘Hongxiangsu’ cultivars, are shown in Figure 1.

### DNA extraction and library construction

The first few young leaves that appeared at the end of the current shoot of each individual were collected at the beginning of the vegetative period of growth. The leaves were immediately frozen in liquid nitrogen and transferred to a −80 °C laboratory freezer. The genomic DNA was isolated using the cetyltrimethylammonium bromide method,^45^ and two libraries were constructed out of the pooled samples for QTL-seq, insertion/deletion (InDel) and simple sequence repeat analyses.

A red-skinned pool was constructed from equal amounts of DNA from 28 red-skinned pears, and a green-skinned pool was constructed from equal amounts of DNA from 27 green-skinned pears. Libraries with 350-bp inserted fragments were constructed using a TruSeq Nano DNA HT Sample Preparation Kit (Illumina Inc., San Diego, CA, USA) following the manufacturer’s protocol and sequenced using an Illumina HiSeq 4000 instrument (San Diego, CA, USA) to obtain 150-bp paired-end reads.

### Sequence data analysis

Quality trimming was conducted to generate high confidence variant calling. The raw reads were processed to obtain high-quality clean reads by (1) removing reads with ⩾10% unidentified nucleotides and (2) removing reads with >50% bases having low Phred quality scores (<5). The Burrows-Wheeler Aligner^46^ was used to align the clean reads from each sample against the public reference genome for pears^47^ using ‘mem 4−*k* 32−*M*’, where *k* is the minimum seed length and *M* is an option used to mark shorter split alignment hits as secondary alignments. The resulting alignment was used to identify SNPs and InDels. Variant calling was performed on all samples using the GATK’s Unified Genotyper 3.3,^48^ and SNPs and InDels were filtered using GATK’s Variant Filtration with appropriate parameter settings (-Window 4, -filter ‘QD<4.0||FS>60.0||MQ<40.0 ‘, -G_filter ‘GQ<20’). Variants exhibiting segregation distortion or sequencing errors were discarded. The ANNOVAR Software Tool (Philadelphia, PA, USA)^49^ was used to align and annotate SNPs and InDels and to determine their physical locations in the genome.

**Figure 2:**
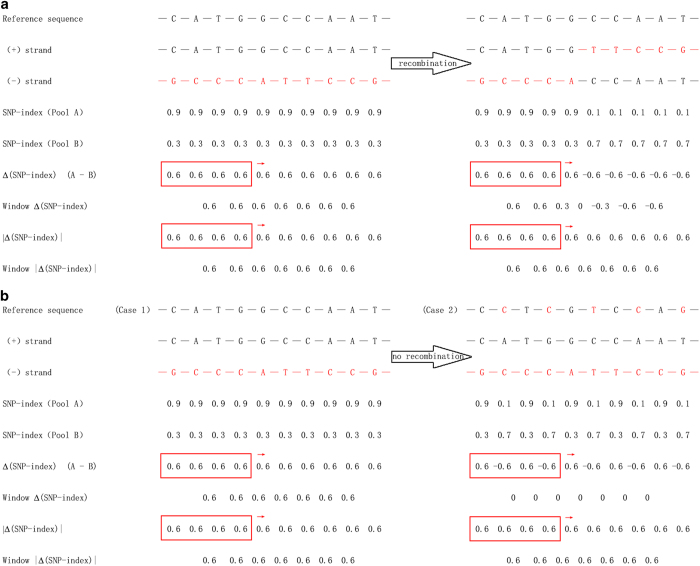
Demonstration of QTL-seq analysis under different scenarios. **a**: Scenario of chromosome recombination in a homozygous crop; **b**: Scenario of reference sequence change in a heterozygous crop. Case 1 in **b**: The ideal condition of using Δ (SNP-index) to locate the correct interval. Case 2 in **b**: The reference sequence is changed. Therefore, it is not possible to use Δ (SNP-index) to locate the trait. The red rectangle boxes indicate the sliding window representing QTL-seq analysis; ⟶ indicates the slide direction of the window; Window Δ (SNP-index) and Window |Δ (SNP-index)| indicates the average value of the Δ (SNP -index) and |Δ (SNP -index)| in the red rectangle boxes, respectively.

The SNP-index was defined as the ratio between the number of reads of a mutant SNP and the number of reads corresponding to the SNP. The index is equal to 1 when all short reads differ from the reference genome and is equal to 0 when all short reads are identical to the reference genome.^36^ In homozygous crops, changes in the SNP-index and Δ(SNP-index) in the offspring pool are usually caused by the exchange and recombination of homologous chromosomes when the QTL-seq analysis of the genome of one of the parents is taken as the reference sequence. Using the Δ(SNP-index) for sliding window analysis, changes in the SNP-index and Δ(SNP-index) of the SNP sites within the candidate interval are consistent within the recombination section, and the candidate section and the different phenotypes correspond to the sign of the Δ(SNP-index) window (Figure 2a). When QTL-seq analysis is performed in heterozygous crops, the switch of the corresponding section of the reference sequence between two homologous chromosomes also results in changes in the SNP-index and Δ(SNP-index) of the offspring, producing an effect similar to that of homologous chromosome exchange, which we refer to as the ‘pseudoexchange effect.’ Site changes in the reference sequence of heterozygous crops are random, which may result in the opposite notation of the Δ(SNP-index) of the adjacent site. The window Δ(SNP-index) would tend to 0, and use of the Δ(SNP-index) for sliding window analysis may miss a portion of the candidate interval (Figure 2b).

In this study, both parental lines belonging to the red-skinned pears were highly heterozygous, which was not ideal for calculating the SNP-index of the DNA pool of the F1 generations because the origin of the SNPs in the target area could not be estimated based on the positive/negative sign of the Δ(SNP-index) window. Therefore, the genome sequence of the ‘Dangshansuli’ ^47^ cultivar was used as the reference to calculate the SNP-index and Δ(SNP-index) between the red- and green-skinned pools to save labor. The average SNP-index of the SNPs in the window was calculated and used for the sliding window plot. Sliding window analysis was performed on the scaffold sequences in the genome file 225117_ref_Pbr_v1.0_chrUn.fa (ftp://ftp.ncbi.nlm.nih.gov/genomes/Pyrus_x_bretschneideri/CHR_Un/) and applied to the SNP-index, Δ(SNP-index) and |∆(SNP-index)| plots with 200-kb windows and 20-kb increments.

Because the SNP-index was based on a reference genome^47^ rather than the genome of the parental line, the origin of each SNP or the linkage around its neighboring region could not be estimated. Positive or negative values of the |Δ(SNP-index)| did not correspond to the phenotype of the parental lines. Reorganization and ‘pseudoexchange’ can change the positive and negative direction of the Δ (SNP-index) but cannot change its absolute value (i.e., |Δ(SNP-index)|). Although use of |Δ(SNP-index)| in a sliding window analysis ignores the positive and negative direction of Δ(SNP-index), it had good positioning ability, especially in locating trait loci in heterozygous crops (Figure 2b). Therefore, the |Δ(SNP-index)| was used as the main parameter to identify the target phenotype. As the length of the pear genetic map is ~1500–2000 cM and the resolution of an initial QTL mapping is typically ~5–10 cM, translating into ~0.5% of the genetic map, the top 0.5% SNP intervals with the highest |Δ(SNP-index)| were selected as candidate gene intervals.

### Mapping to linkage groups

The location of the scaffolds containing candidate gene intervals was determined using the high-density genetic map of ‘Bayuehong’×‘Dangshansuli’ published by Wu *et al.*^50^ Use of the congruent relationships between the scaffold names in the file ‘scaffold_names’ (ftp://ftp.ncbi.nlm.nih.gov/genomes/*Pyrus*_x_*bretschneideri*/) provided the ability to determine the linkage groups of the scaffolds that contain candidate intervals based on their SNP markers in ‘Supplementary Table S4’.^50^ This information was compared with the corresponding positions for the red-skinned trait loci in Asian pears reported by Wu *et al*.^50^ to confirm the results.

### Linkage verification

To verify the accuracy of the candidate interval from QTL-seq using linkage analysis, several primers for InDel and simple sequence repeat markers within the candidate intervals were designed using the Primer3 Software (Tartu, Estonia).^51^ The primers for these markers were then screened on nine red-skinned and seven green-skinned genotypes from the red-skinned and green-skinned pools, respectively. Twelve markers that were polymorphic between the nine red-skinned and seven green-skinned genotypes were selected and successfully amplified in the F1 population resulting from the ‘Mantianhong’×‘Hongxiangsu’ cultivar cross. The polymerase chain reactions were carried out in a final reaction volume of 20 μL, containing 20 ng of DNA, 1× polymerase chain reaction buffer (Mg^2+^ plus), 0.2 mmol/L of each dNTPs, 6 pmol of each primer and 0.5 U *Taq* DNA polymerase (TaKaRa Biotechnology, Dalian, China), using the following amplification program: initial denaturation for 3 min at 95 °C, 30 cycles for 30 s at 95 °C, 30 s at 55 °C and 45 s at 72 °C, and a final 10-min extension at 72 °C. The amplicons were evaluated using 8% non-denaturing polyacrylamide gel electrophoresis. Joinmap 4.0^52^ was used to perform the linkage analysis. A logarithm of odds score of 4.0 was set for grouping, and the genetic distances were calculated using the Kosambi function.

## Results

### Inheritance of the red/green-skinned phenotype in Asian pears

The red- and green-skinned phenotypes segregated in the F1 populations of the two Asian parental lines. An analysis of the 2014 and 2015 data indicated that there were 117 and 182 red- and green-skinned pear individuals, respectively, among the 299 ‘Mantianhong’×‘Hongxiangsu’ F1 hybrids. Among the 310 ‘Yuluxiang’×‘Mantianhong’ F1 hybrids, there were 148 and 162 red- and green-skinned individuals. The segregation data are presented in Table 1.

### Genes responsible for the red/green-skinned phenotype narrowed down to a 1.86-Mb interval using QTL-seq

Illumina high-throughput sequencing resulted in 78 220 374 and 93 410 888 reads from the red-skinned (19.65×depth coverage or 94.74% coverage) and green-skinned (22.98×depth coverage or 95.15% coverage) DNA pools, respectively. The resulting reads were aligned to the pear reference genome,^47^ and SNPs were identified. An SNP-index was calculated for each identified SNP. Low-quality SNPs with an SNP-index value <0.3 and a read depth <7 or those without an SNP-index in one of the F1 hybrid pools were excluded to decrease the influence of sequencing and alignment error. A total of 2 280 509 SNPs were identified within the two DNA pools.

**Figure 3:**
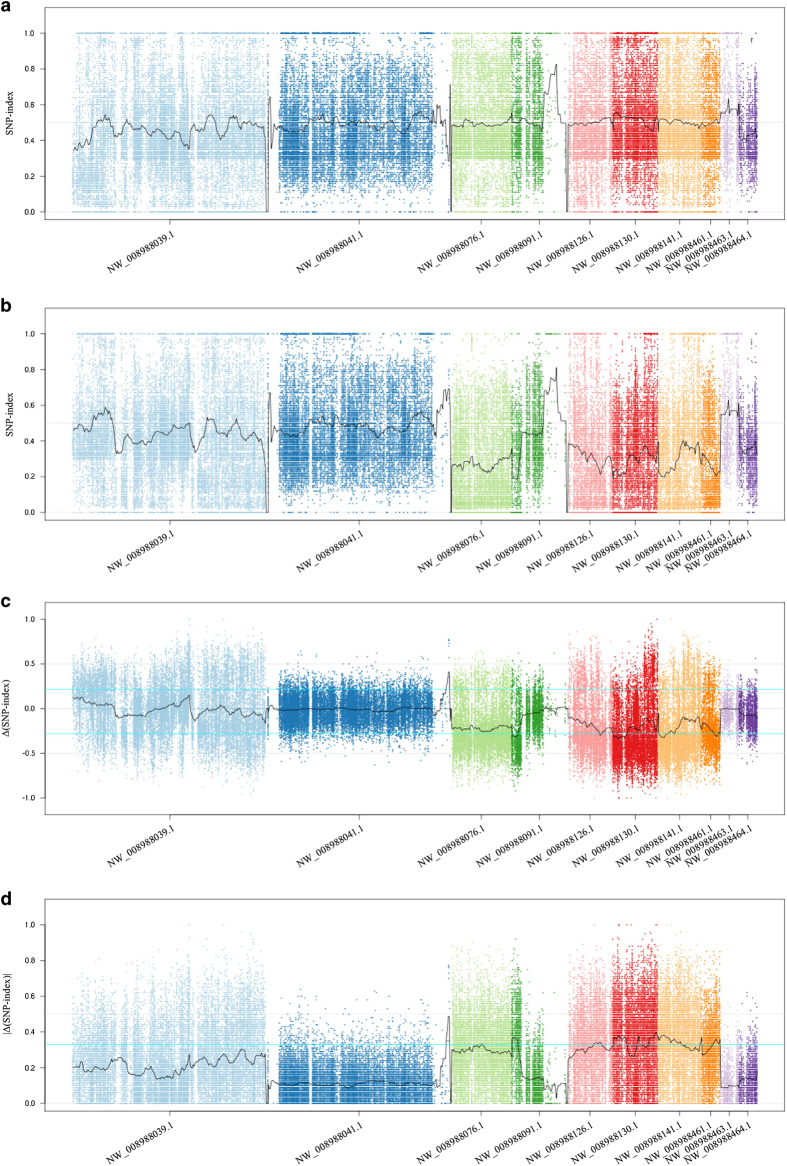
SNP-index graphs of the red pool (**a**), green pool (**b**), Δ(SNP-index) (**c**) and |Δ(SNP-index)| (**d**) derived from the QTL-seq analysis. The X-axis indicates the position of the ten scaffolds and the Y-axis indicates the SNP-index.

The top 0.25% and lowermost 0.25% threshold lines for the ∆(SNP-index) plot (Figure 3) were set at 0.215 and −0.280, respectively. The threshold line for the top 0.5% |∆(SNP-index)| plot was set at 0.33, which was greater than the 0.215 and 0.280 values previously indicated. This was primarily due to the inability to obtain allele linkage information of the adjacent SNP loci from the highly heterozygous pear genome. Therefore, the plus or minus sign of the ∆(SNP-index) may have been opposite to the actual linkage. This problem eventually resulted in an offset within the window through averaging of the ∆(SNP-index) values, and the ∆(SNP-index) value of the window tended to be zero (Figure 2).

The candidate intervals derived from thresholds of −0.280 and 0.215 based on the ∆(SNP-index) resulted in the partial overlap of some intervals from the |∆(SNP-index)| using a threshold of 0.33. Based on the |∆(SNP-index)| method, the candidate interval was 2.18 Mb. However, based on the ∆(SNP-index) method, the candidate interval was 1.72 Mb, indicating a 1.1 Mb overlap. Although both methods had a similar distribution on some scaffolds, the candidate interval estimated by the ∆(SNP-index) method was relatively smaller, and most intervals were in the subset of the |∆(SNP-index)| method. The influence of the ‘pseudoexchange effect’ caused many of the intervals predicted by the |∆(SNP-index)| method and verified in the linkage analysis as candidate intervals to be missing in the ∆(SNP-index) method results. This was the case for the intervals located between 500 001 and 580 000 bp and between 760 001 and 960 000 bp on scaffold NW_008988130.1 and between 880 001 and 940 000 bp on scaffold NW_008988141.1 (Figure 3), which indicated that the |∆(SNP-index)| method was much better than the ∆(SNP-index) method for locating trait loci in heterozygous crops (Figure 2b). The size of the interval estimated by the |∆(SNP-index)| method was reduced from 2.18 to 1.86 Mb after filtering the intervals, with only a few SNPs that were widely separated (Table 2).

### Linkage group location of the major number of scaffolds

Most scaffolds containing candidate intervals were located on the bottom portion of the fifth linkage group (LG5). The exceptions that did not map to a linkage group included NW_008988041.1, NW_008988581.1 and NW_008989660.1. The remaining nine scaffolds, NW_008988039.1 (scaffold1.0.1), NW_008988076.1 (scaffold40.0), NW_008988091.1 (scaffold56.0.1), NW_008988126.1 (scaffold92.0), NW_008988130.1 (scaffold97.0.1), NW_008988141.1 (scaffold110.0), NW_008988461.1 (scaffold430.0.1), NW_008988478.1 (scaffold448.0.1) and NW_008989715.1 (scaffold1650.0), were adjacent to each other on LG5 and concentrated in the area between 111.9 and 177.1 cM (Table 3). These results indicate the accuracy of the modified QTL-seq analysis.

### Linkage analysis to narrow the candidate interval

**Figure 4:**
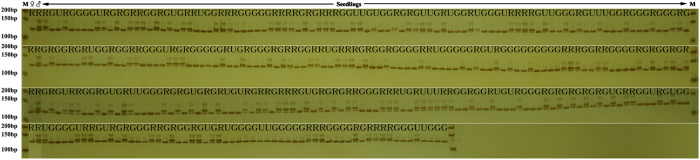
PCR amplification of the InDel marker, ZFRI130-16 in the F1 individuals of the ‘Mantianhong’×‘Hongxiangsu’ cross. Results conform to the CP pattern nn × np. M: DL2000 Marker, ♀: ‘Mantianhong’, ♂: ‘Hongxiangsu’, R: Red skin phenotype, G: Green skin phenotype, U: Unknown phenotype.

**Figure 5:**
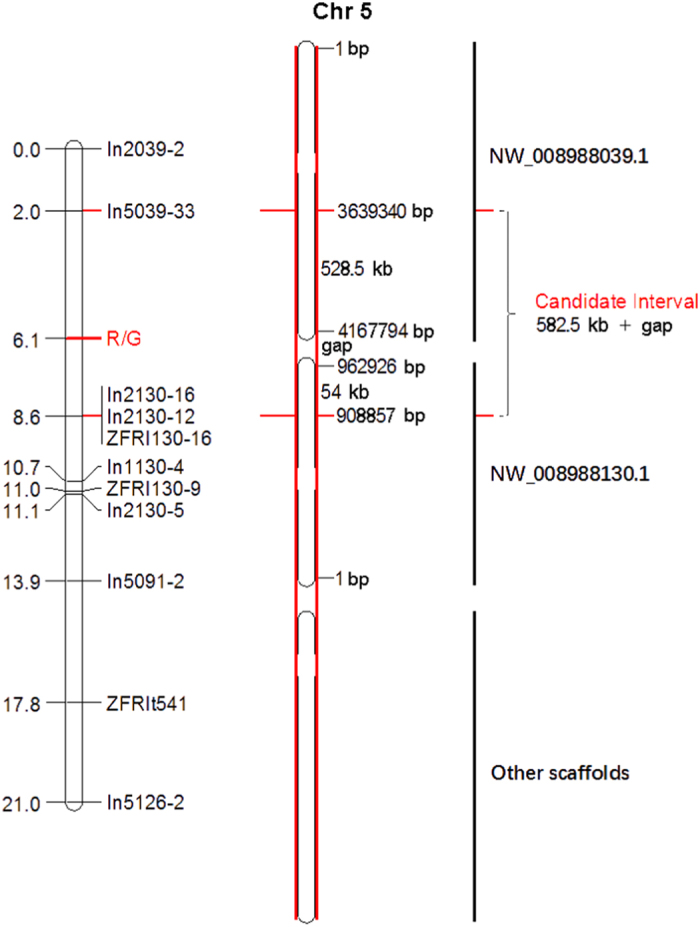
Molecular markers and the red/green skin locus on LG5. The left one is the result of R/G locus linkage position, the middle one is the diagram of the chromosome, the black vertical bar on the right indicates the corresponding position of the scaffolds on the chromosome map, the red line on the right indicates R/G locus and the candidate interval.

InDel (231) and simple sequence repeat (55) primer pairs (Supplementary Table 1) were used to screen for polymorphisms in the candidate intervals. Twelve primer pairs produced clear bands and performed the best in red/green skin identification. Therefore, these 12 pairs of primers were used to perform a linkage analysis using the F1 hybrid individuals resulting from the ‘Mantianhong’×‘Hongxiangsu’ cross (Figure 4). Nine individuals were determined to be non-parental-line cross-hybrids and were removed from the data set, and 339 F1 hybrids were maintained in the subsequent analyses. A linkage group with a total length of 21.0 cM was obtained, containing 11 molecular markers and red/green skin trait loci (Figure 5 and Table 4). Three markers, In2130-12, ZFRI130-16, In2130-12 and In2130-16, were located near the red/green (R/G) locus at a genetic distance of 2.5 cM. The markers also showed an accuracy rate of >90% in 310 fruited individuals of ‘Yuluxiang’ and ‘Mangtianhong’ and accuracy rates of 80.85%, 85.11% and 97.87%, respectively, in 47 plants (34 accessions derived from the male parent ‘Hongxiangsu’ and 13 cultivars) in an additional validation (Xue, 2016, unpublished data). These could be used as the molecular markers for breeding selection of red-skinned fruit in Asian pears. Marker In5141-1, which could accurately distinguish the fruit skin color of 89.30% of the offspring, was not located in this linkage group because its segregation did not conform to the ‘CP’ population model (Supplementary Figure 1).

The R/G locus in Asian pears was located between marker In5039-33 and markers ZFRI130-16, In2130-12 and In2130-16, which were identified in scaffolds NW_008988039.1 (scaffold1.0.1) and NW_008988130.1 (scaffold97.0.1), respectively. These two scaffolds were adjacent to each other on the bottom of LG5. Markers In2039-2 and In5039-33 from scaffold NW_008988039.1 and markers ZFRI130-16, In2130-12 and In2130-16 from scaffold NW_008988130.1 determined the direction of the two scaffolds. Therefore, the R/G locus should be in the 582.5 -kb candidate interval comprising two subintervals at the bottom of LG5: a 528.5-kb interval from 3 639 340 to 4 167 794 bp in scaffold NW_008988039.1 and a 54-kb interval from 908 857 to 962 926 bp in scaffold NW_008988130.1 (Figure 5 and Table 4). This candidate interval also includes a gap of unknown size between scaffolds NW_008988039.1 and NW_008988130.1. The markers in the same scaffold were staggered, and there was no linear relationship between the physical position and the map position. Therefore, the range of position of the red/green skin trait on LG5 of Asian pear could not be narrowed further.

Based on the reference pear genome, 81 gene models were predicted in the candidate 582.5 kb genomic region (Supplementary Table 2). Among these, 10 were not annotated with a known function, and 71 genes had a predicted gene function. No structural genes associated with the anthocyanin biosynthetic pathway, such as *PAL*, *CHS*, *CHI*, *DFR *(dihydroflavonol 4-reductase), *ANS* and *UFGT*, were found within the candidate region. The MYB transcription factor that forms the MBW complex, the basic helix–loop–helix (bHLH) and the WD40 classes were not detected within the candidate region. Several genes, including the NAC domain class transcription factor, the WRKY domain class transcription factor and ABC transporter B family member 9-like, all of which are related to anthocyanin synthesis and accumulation, were found in the candidate region but require further identification and confirmation.

## Discussion

Whole-genome sequencing is a fast and cost-effective approach for identifying genetic variants.^53–56^ The pool sequencing (Pool-seq) strategy, which combines BSA and whole-genome sequencing, can rapidly identify linked genomic regions to target trait loci in mutation discovery^57,58^ and has been used in model organisms including *Arabidopsis thaliana*,^59,60^ nematodes,^61,62^ zebrafish^63–65^ and mice.^66^ Most studies using Pool-seq identified regions of mutations based on allele frequency and SNP density, but the SNP-index approaches used in rice were simpler and more efficient.^35–37^ The parental lines used in Pool-seq studies have typically been homozygous, and thus the linkage relationships among SNPs have been unambiguous. Variant regions have been identified by determining which SNP genotypes originated from each parent. A few studies used Pool-seq without a parental genome sequence because the genome in question was highly heterozygous or for other reasons. For example, Hill *et al*.^32^ used the Euclidean distance to reduce the noise inherent in RNA sequencing data sets when identifying recessive mutations in zebrafish without parental strain information. In the present study, we used the absolute value of ∆(SNP-index) to shield against background noise caused by the highly heterozygous parental lines and multiple levels of sampling. Both the introduction of the Euclidean distance and |∆(SNP-index)| reduced the dependence of Pool-seq on parental genome sequences and may thus be used in a wider scope of applications.

QTL-seq has been successfully used to conduct a BSA analysis of a segregated population of homozygous parental lines with opposite phenotypes. Calculation of the ∆(SNP-index) for the DNA pool from filial generations based on the genome of one of the parental lines revealed the parental origins of the SNPs in a hybrid pool, which are easy to relate to the target phenotype.^36,39,41,42,67^ In our study, we were not able to identify the linkage among filial and parental SNPs because the parental genomes were highly heterozygous. Estimating the parental origins of the SNPs according to the ∆(SNP-index) could generate errors. The positive or negative sign of the ∆(SNP-index) may have been opposite to the actual condition, resulting in a ∆(SNP-index) with an opposite direction offset within the window, with the ∆(SNP-index) value of the window approaching 0, reducing the QTL-seq detection sensitivity (detectability) of the candidate interval with target phenotypes (Figure 2). For example, the three nearest markers to the R/G locus were located within the 760 001 to 960 000 bp interval on scaffold NW_008988130.1, but this interval was not predicted by the ∆(SNP-index).

If candidate intervals are dispersed on the scaffold, the target trait intervals are interlaced with non-target trait intervals, which decreases the average value of the |∆(SNP-index)| in a window. In the current study, predicting the interval linked with target traits proved impossible. The downstream 2 500 000 bp section on scaffold NW_008988039.1 fluctuated widely, and the peaks were near the threshold. Therefore, this section could not be the candidate interval (Figure 3d). To avoid missing the candidate interval, we designed primers for the target interval and conducted polymerase chain reaction analyses. A strong linkage relationship was found between relevant sites in this interval and the R/G locus in Asian pears (Figure 5). Accordingly, we used |∆(SNP-index)| instead of the ∆(SNP-index) to conduct the QTL-seq analysis and successfully obtained the interval linked to the red/green skin phenotype. Therefore, we believe that the analytic strategy used in the present study could be expanded to identify the loci for other traits.

Dondini *et al*.^13^ analyzed seven crosses in European pears with full red bud sport and green-skinned phenotypes. Their study showed that the former phenotype in European pears was controlled by a single dominant gene, *Red*. This gene was located between two amplified fragment length polymorphism markers on the LG4 of the ‘Max Red Bartlett’ map, which was 13.5 cM from marker E31M56-7 and 18.2 cM from marker E33M48-5.^13^ In ‘Bayuehong,’ a red-blushed cultivar derived from a cross between ‘Clapp’s Favorite’ and ‘Zaosu,’ four QTLs for red skin from ‘Clapp’s Favorite’ were on LG4, LG13 and LG16.^50^ The *Red* gene was located at 64.0 cM of LG4, whereas Pyb04_016, a QTL for red skin from ‘Clapp’s Favorite,’ was at 4.8 cM, indicating that they were far apart. The present study used the Asian pear cultivars ‘Mantianhong’ and ‘Hongxiangsu’ as parental lines. Their red skin trait was inherited from the ‘Huobali’ sand pear cultivar and the ‘Korla Xiangli’ Xinjiang pear cultivar. We located the R/G locus in a 582.5 -kb candidate interval at the bottom of LG5. This finding is not in agreement with the findings reported by Dondini *et al*.^13^ and Wu *et al*.^50^ These varying mapping results indicate that there are different mechanisms for the control and regulation of the red skin trait in Asian and European pears and different types of red-skinned phenotypes within European pears.

In the hybrid group of the red-skinned Asian pears, the coloration of the progeny showed the character of the alternative quality traits. Volz *et al*.^29^ analyzed the segregation of red characters in the progenies of 10 Asian pear hybrids and suggested that it could be controlled by two dominant gene loci, which is consistent with the findings in apples.^68^ In our study, the segregation ratios of red to green were 2:3 (Mantianhong×Hongxiangsu) and 8:9 (Yuluxiang×Mantianhong), which were lower than the minimum segregation ratio (9:7, with AaBb and AaBb genotypes) in the hypothesis assuming two independent dominant gene loci. The ratio of red to green is as low as 1:1 only when the two loci are closely linked or their Ab|aB alleles are cosegregated, the segregation type of these two loci is hk×hk and the homozygous and heterozygosity genotypes of progeny correspond to the green- and red-skinned phenotypes, respectively. However, in this study, the Asian pear red skin trait loci were in a continuous interval on the fifth chromosome, and the segregation types of three closely linked molecular markers with red-skinned traits were nn×np, not hk×hk.

Our findings support the hypothesis that the trait is controlled by a single dominant gene that tends toward green-skinned segregation. This same confusion was observed in apples. At present, only one locus has been found to control the red-skinned trait in apples.^69^ Deviation from Mendel's genetic separation in the hybrid progeny of the red-skinned Asian pear with bias toward green separation appears to be a common phenomenon ^28,29,55^ and may be a result of the linkage of red skin trait loci with lethal or semilethal gene loci.^29^ A higher distorted segregation ratio of molecular markers was also detected when we identified the hybrid progeny of ‘Mantianhong’ and ‘Hongxiangsu’.^70^ Additional work is needed to fully understand the genetic regularity of the red-skinned trait of Asian pears, including analysis of hybrid progeny segregation and genotypes, development and application of molecular markers, determination of the mechanism of distorted segregation and validation of gene function.

The color area and degree of redness in the skin of Asian pears are influenced by environmental conditions and show the characteristics of quantitative traits. The structure gene and transcription factor gene of the anthocyanin biosynthetic pathway play important regulatory roles in this process. However, it is unclear if the quality trait genes that control the coloration of the red-skinned pear are derived from the structural gene or transcription factor gene of the anthocyanin biosynthetic pathway, and no such genes were found in the known gene model within the candidate intervals in this study. Therefore, it is not possible to exclude the presence of a gene that acts as a switch in the coloration of red-skinned pears upstream of anthocyanin biosynthetic pathway-related genes or transcription factors. The most interesting candidate genes identified within the region containing the R/G locus were the NAC domain class transcription factor, the WRKY domain class transcription factor and the ABC transporter B family member 9-like gene, which have all been associated with anthocyanin synthesis and accumulation. The NAC transcription factors play important roles in plant stress responses, flowering and secondary cell-wall biosynthesis.^71,72^ An expression sequence tag encoding the transcription factor NAC domain protein was found to be selectively induced by cold in blood oranges but not in common oranges.^73^ A report on blood-fleshed peaches suggested that the heterodimer of two NAC transcription factors (BL and PpNAC1) can activate the transcription of *PpMYB10.1*, resulting in anthocyanin accumulation, and that *BL* is the key gene for the blood-flesh trait in peaches.^74^ Interestingly, there are two R2R3 *MYB transcription factor 10* genes in scaffold NW_008988130.1 near the candidate region (listed in Supplementary Table 2). The relationship between the two R2R3 *MYB* genes and the NAC transcription factors in the candidate intervals merits additional investigation.

Although WRKY transcription factors play an important role in the plant stress response and hormone signaling,^75^ some WRKY family genes play an important role in regulation of anthocyanin accumulation. For example, the WRKY transcription factor AtTTG2 has been reported to regulate the accumulation of proanthocyanidins and anthocyanins in *A. thaliana*.^76,77^ The TTG2-like homolog protein VvWRKY26 is also involved in the regulation of vacuolar transport and flavonoid accumulation in grape berries.^78^ In addition, a study on *A. thaliana* photomorphogenesis and root development revealed that two ATP-binding cassette transporter proteins, AtMDR1 and AtPGP1, are required for normal accumulation of anthocyanins.^79^ The results of the present and previous studies clearly indicate the need for additional and more detailed work to identify candidate genes associated with anthocyanin synthesis and accumulation. Molecular analyses should be conducted on more taxa. Gene sequences and expression should also be compared between red- and green-skinned genotypes. These additional studies will elucidate the genetic control and regulatory processes responsible for the red-skinned trait in Asian pears.

## Figures and Tables

**Table 1:** Segregation of the red/green-skinned trait in individuals from the two crosses in this study

*Group*	*Year*	*Fruited individuals*	*Red individuals*	*Green individuals*	*Red:green*
‘Mantianhong’×‘Hongxiangsu’	2014	177	80	97	4:5
	2015	279	111	168	2:3
	Total	299	117	182	2:3
					
‘Yuluxiang’×‘Mantianhong’	2014	189	101	88	8:7
	2015	262	121	141	6:7
	Total	310	148	162	8:9

**Table 2:** Potential chromosomal regions of red/green-skinned trait loci in Asian pears

*Scaffold*	*Start*	*End*	*Region length*
NW_008988041.1	3 800 001	3 880 000	80 kb
NW_008988076.1	640 001	660 000	20 kb
NW_008988091.1	1	140 000	140 kb
NW_008988126.1	560 001	720 000	160 kb
NW_008988130.1	1	300 000	300 kb
NW_008988130.1	500 001	580 000	80 kb
NW_008988130.1	760 001	960 000	200 kb
NW_008988141.1	1	500 000	500 kb
NW_008988141.1	880 001	940 000	60 kb
NW_008988461.1	240 001	400 000	160 kb
NW_008988478.1	300 001	400 000	100 kb
NW_008988581.1	300 001	320 000	20 kb
NW_008989660.1	1	20 000	20 kb
NW_008989715.1	1	20 000	20 kb
			
Total			1.86 Mb

**Table 3:** Linkage groups of scaffolds containing the candidate intervals

*RefSeq accession file*	*Genome center name*	*Linkage group (Wu et al. *^50^)
NW_008988039.1	Scaffold1.0.1	LG5
NW_008988041.1	Scaffold3.0	LG15
NW_008988076.1	Scaffold40.0	LG5
NW_008988091.1	Scaffold56.0.1	LG5
NW_008988126.1	Scaffold92.0	LG5
NW_008988130.1	Scaffold97.0.1	LG5
NW_008988141.1	Scaffold110.0	LG5
NW_008988461.1	Scaffold430.0.1	LG5
NW_008988478.1	Scaffold448.0.1	LG5
NW_008988581.1	Scaffold543.0.1	Unknown
NW_008989660.1	Scaffold1606.0	Unknown
NW_008989715.1	Scaffold1650.0	LG5

**Table 4:** Twelve R/G locus-linked markers in Asian pears

*Locus*	*Primer sequences (5′→3′)*	*Scaffold*	*Scaffold position*	*Mapping position*
In2039-2	F: CCTTACCATCTCAAACTCCA R: GTCAAAGGGTTACTGGTTCA	NW_008988039.1	3 280 367	0.0
In5039-33	F: ATTTGGTGAGATCATCATGG R: TAACTTCACACGGACTTCCT	NW_008988039.1	3 639 340	2.0
R/G	—	—	—	6.1
In2130-12	F: TTGGAGATGTTACTGTGACG R: GATTACATGCATTGACGTTG	NW_008988130.1	908 857	8.6
In2130-16	F: AATAGAAATTTAACACCTACGC R: ACATATAGCACCCTTAGGAAA	NW_008988130.1	920 222	8.6
ZFRI130-16	F: CACTTTCGAAGCCAACAAAT R: GGGCAAACTGGGAAACTG	NW_008988130.1	922 094	8.6
In1130-4	F: GGCATTACTCTAATCTTCAATC R: AGAAACCCGAGACTAATCAT	NW_008988130.1	154 093	10.7
ZFRI130-9	F: ACCAGCGTAGTTTGATAAGA R: AAGGTCGTCAAGTGAGAAG	NW_008988130.1	435 305	11.0
In2130-5	F: CCGATCCGTAAGTTCCTAT R: TCTAAAGATTGCCCAGGATA	NW_008988130.1	113 643	11.1
In5091-2	F: TTACGAGGGGAAATCAATTA R: GAAATTGAAATTGCTCGTCT	NW_008988091.1	15 549	13.9
ZFRIt541	F: AAAAACCGAAAACAAAAACCTC R: CGGAGCCCTGTGCTACTAAT	NW_008988130.1	916 907	17.8
In5126-2	F: GCTATTTTGAAATGCCGTAT R: TCCTTCCCAAGTTATTTGAA	NW_008988126.1	50 318	21.0
In5141-1	F: CAAAACAGGAAGACACATGA R: AACCATGCGTTGACTAAAAT	NW_008988141.1	7013	—
