# Supplementary figures and images for: Interval mapping for red/green skin color in Asian pears using a modified QTL-seq method
Source: Hortic Res. 2017 Oct 4;4:17053–. doi: 10.1038/hortres.2017.53 (PMC5674137; doi:10.1038/hortres.2017.53)

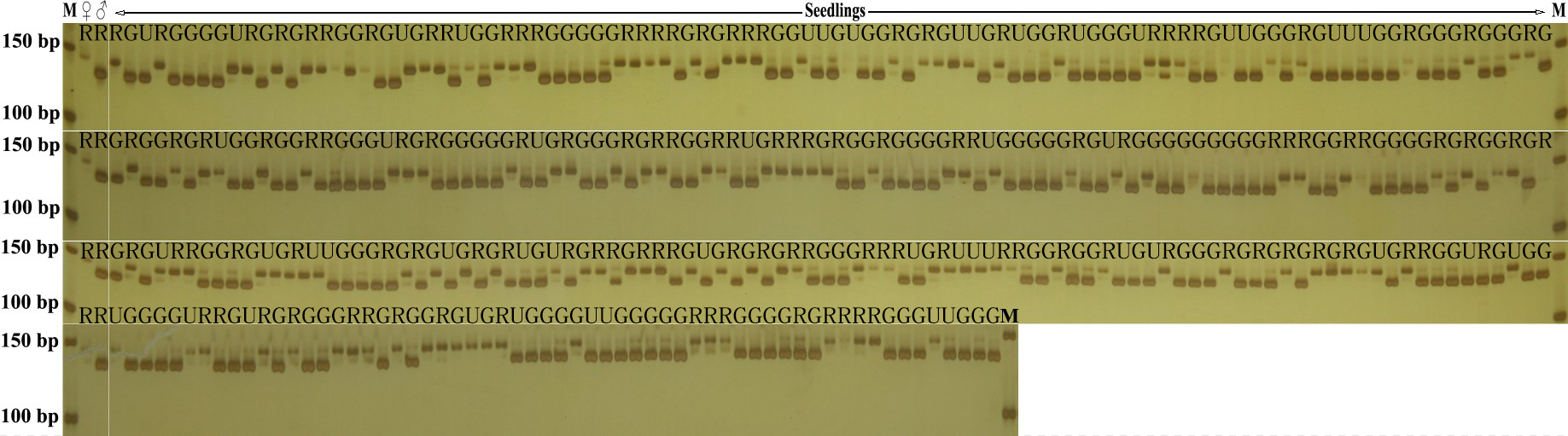

Supplement: Supplementary Figure 1 [file hortres201753-s1.docx]
