# Supplementary material for: Interval mapping for red/green skin color in Asian pears using a modified QTL-seq method
Source: Hortic Res. 2017 Oct 4;4:17053–. doi: 10.1038/hortres.2017.53 (PMC5674137; doi:10.1038/hortres.2017.53)
Supplement: Supplementary Table 1 [file hortres201753-s2.docx]

Supplementary Table 1. Primers used to amplify the candidate intervals examined in this study.

| **Site** | **Marker type** | **Forward primer (5’→3’)** | **Reverse primer (5’→3’)** |
| --- | --- | --- | --- |
| In1130-1 | InDel | AAATCCGATTCCATAATTCC | ACACATATCCCACCATTTGT |
| In1130-2 | InDel | GGACTCATTTCGTTCTTTTG | CGAAAATGGTAACATCGTTT |
| In1130-3 | InDel | TGTTGCAATGTCTAATCCAA | CAATTTCCAAAATGGTAAGC |
| In1130-4 | InDel | GGCATTACTCTAATCTTCAATC | AGAAACCCGAGACTAATCAT |
| In1130-5 | InDel | CGTTTGTCAAGTTCATGTTG | ACTAAACCTCCATACGAGCA |
| In1130-6 | InDel | ATTTAATTCGAGGTGCATTC | TGTAATTTAAGCATGGGTCC |
| In2039-1 | InDel | ATACACTTTTCTTAGCGCGT | TCTCTCTTTCTTCTCCATGC |
| In2039-2 | InDel | CCTTACCATCTCAAACTCCA | GTCAAAGGGTTACTGGTTCA |
| In2039-3 | InDel | CAGACTCTCATCCTTGAAGC | TTATTTCCCAGAAATTGCAC |
| In2039-4 | InDel | GGGTTTTGGAAATTCTTGTA | ATTCCACATCCAAAACAAAG |
| In2039-5 | InDel | CTGTGTATGTGCTTTGTGCT | TACGAGAGAGAGGTGTTGGT |
| In2039-6 | InDel | ATCGCATACGGATATAATGC | AAAATAATTCCCTCTCTGCC |
| In2039-7 | InDel | GTAACTTGTTTTCGGGAATG | ACCAATAATGCTCTTTGGAA |
| In2126-1 | InDel | TCGAAGCTCAAGTTTCATTT | TAGCACGTGGATAAATTGTG |
| In2130-1 | InDel | AGATACTTTGCTGAGAACGG | ACTTTGTTTCAAGCATTTGG |
| In2130-2 | InDel | CCAAATGCTTGAAACAAAGT | TTGTGTCAAAATTCAAGGTTT |
| In2130-3 | InDel | AAGGTGGTAAGTTGGCTGTA | CAGTAACAAGGGGTGAAAAA |
| In2130-4 | InDel | ATCCAAACCACAAACTCAAC | GTAGCATCCATGCCTAAAAC |
| In2130-5 | InDel | CCGATCCGTAAGTTCCTAT | TCTAAAGATTGCCCAGGATA |
| In2130-6 | InDel | GGAGTTTTGATGGAACTTGA | TTGTAACATCCCACATAGCC |
| In2130-7 | InDel | AGAGAAAATTTGATGAGTATCG | CATTCTTCTCTCTCCTCTTCTT |
| In2130-8 | InDel | CCAAGAAAATTTAAGAAGAGGA | TTGGCACATTCTCATTACAC |
| In2130-9 | InDel | TAAGTAGGATGCGCCTTATT | CGGTTTTGATCACTGATTTT |
| In2130-10 | InDel | AGCCTTCTACACTCAAGCAG | TAATCAGTTAATAGGCCGGA |
| In2130-11 | InDel | CGTGTTCTGCAAAATGTCTA | TCTTGAAGGGTCTCAATGTT |
| In2130-12 | InDel | TTGGAGATGTTACTGTGACG | GATTACATGCATTGACGTTG |
| In2130-13 | InDel | CTTTCCAGCAATCTCTGTTC | AGGGGAATTTTGTCTCAAAC |
| In2130-14 | InDel | CCAACACTGGACCTACATTT | CTTCTCAAAGGCCATTCTAA |
| In2130-15 | InDel | CAATAAACAATTTCCCATCC | TTGGAGACTCAAAAACAATTT |
| In2130-16 | InDel | AATAGAAATTTAACACCTACGC | ACATATAGCACCCTTAGGAAA |
| In2130-17 | InDel | TTTAGTCGACACGTTAGCAA | ACCAAAATTTCCCGTTTACT |
| In2130-18 | InDel | TTGTGGGATTTTGAGATCAT | CGAGACCTACCTTCATCTTG |
| In2130-19 | InDel | TGATATGTTAGTTCACAGTTCG | AGCTTTGAATAACTTCCAGG |
| In2130-20 | InDel | GACTAAAGTACGCTGCTGCT | AAACAAATTTGGAGCTTTGA |
| In2141-1 | InDel | CTGGCACATTCCATTACTTT | TTCATGAGCTTTCTGATCCT |
| In2141-2 | InDel | TGCCTGAGGTCTGTATTTCT | CTCCCATATGTGTTTTCGAG |
| In3068-1 | InDel | TCGAGGTAATTTTCAGAGGA | AGTTGAAAGATCTGAAGCCA |
| In3068-2 | InDel | CATGTCAGCTGTGGTCTCTA | GTCCTTATTGCAGAATGAGC |
| In3068-3 | InDel | GGATGTCCCGATTCATATAG | ATACACACACACACACACACA |
| In3068-4 | InDel | GTGAGTCTCAGAGTAACCGC | TCTTTGTGCAAAAGCATCT |
| In3068-5 | InDel | AACTCTTGGGTGTTTCTCAA | GCCATAGCTTCACACCTAAC |
| In3068-6 | InDel | GGTGAGTCTTCCTCCTTCTT | TCTGGAAAGTGAGAATGGTC |
| In3068-7 | InDel | CCAACAAAATTTTCGTCTTT | ACCTCCCCTATTCCAACTAA |
| In3068-8 | InDel | CCACCAATTTTGTCATCATA | GGCATCTTCTTCTTCTTCTTC |
| In3068-9 | InDel | CTACAATCTCTGTGTGGGGT | AATGAGGGTTTGATCAGTTG |
| In3068-10 | InDel | AATTTCACGTGATAAAGCAT | AAAATAACCATGTTATCATTCA |
| In3068-11 | InDel | CAGAAGGATCGTCTTCTTGA | GGTCAATACTTGCATATGGG |
| In3126-1 | InDel | CTATATTGGGACGAAAATGC | CCATAGATGTAAAGGGAACCT |
| In3126-2 | InDel | AAAGGCAAGTTATTTTGGGT | GAAAGAGAATAGCCACTCACA |
| In3126-3 | InDel | ACATTGCAGTCATAGTTTCG | ACAACACTCGTCACTTATGC |
| In3126-4 | InDel | TTTTTCTGGTATGTATTGGC | CGAATGAATTAGTATGGTTGT |
| In3130-1 | InDel | TGGGAGGCCTTATGATACTA | GCTGCAAATCCCTTATTCTA |
| In3130-2 | InDel | TACGTTTGCCCTAAGAGATT | CATTTCCAGTGATCCAAAGT |
| In3130-3 | InDel | AGCATGATGGGTTTGATTAG | CCGCTATTATCCAAAATGAC |
| In3130-4 | InDel | TGAAAACTCTCGACAACCTT | CTTATTTTACGGCTCCAGTG |
| In3130-5 | InDel | GATTTTCGAGTCTTTGTTGG | TCTAGAGCCAAAAGACAAGG |
| In3130-6 | InDel | GCCGAAGCTAGAGAATATGA | TTGACGGTAGATTCTTCAGG |
| In3141-1 | InDel | CATAATTTCTCCGCATATCA | AGTGGACCCCTTCTAAACTT |
| In3141-2 | InDel | GTAACCGCACAAATAGAAGG | TTTTTGTTGTGTTCCTTTCC |
| In3141-3 | InDel | GCATCTTTGTTGAATAACCC | TTAAAGTTCCATGAGATGGG |
| In3141-4 | InDel | AGGATCTCTTTCGAAAACTGT | TTTTGGAAGTGAAATAGTGAGA |
| In3141-5 | InDel | GATGAGCACTAACCTCTTGC | ACACAAAGAGAAGAGAAGCG |
| In3141-6 | InDel | TTTAGCGAATATCGGGTTTA | AGAACAAGCAAAGTTTACGC |
| In3141-7 | InDel | ACTGGAACCTGCAAGAACTA | TGAACAAAGAAATCATTAACCA |
| In3333-1 | InDel | CGAATTGCAAGTTCAATAAA | GTGTGTGTGTGTGTGTGTGT |
| In3333-2 | InDel | CCTCTTCAAGCGAGCTACTA | GAGCACGTCATTCTTCAACT |
| In3333-3 | InDel | TTAGCTTTACGGCATCTTGT | GTGATGTGCAAATGTAATGC |
| In3333-4 | InDel | ACGAGGAACGATTCTTCATA | AGCATCTCTTTAACAAGCCA |
| In3333-5 | InDel | GATCGAGAAAGGGATGATAC | AGATGGAGACTTGAAATCCT |
| In3333-6 | InDel | TGATGAAAATAAACACACCG | AAGTGATTTGGAGCTTTCAC |
| In3333-7 | InDel | GTGCAACAAAAGAAGAAAGC | AAAGTCGTTGCCAAATAAGA |
| In3660-1 | InDel | CCAAATTTAGACCCACATGA | AATAATTGAGGGAGGACTGC |
| In3660-2 | InDel | GTGCTATGTAACTTTTGCCC | ACCCTTGACAACGACTAATG |
| In3660-3 | InDel | TATCAAATTCATCGTGCGT | TGTATTTTGGACCACTTTCC |
| In3660-4 | InDel | GGCAGGTCCCTCAATTAT | CCATCTCCAGTTTGGTAGTT |
| In3660-5 | InDel | TAAATAACTTTTGCCCAAGC | TCTTGTACCCTTGACAACAA |
| In3886-1 | InDel | CAAACCACATCTCAGAATCC | ATCTTTGACCGATGATTGAC |
| In3886-2 | InDel | CAACCACGAGGTTAGAAAAG | AAGTCTCTCTCTCCCTCACC |
| In3886-3 | InDel | ACTGCAGATTTACCCTCTGA | ACAAAAATTTGGATGGAATG |
| In3886-4 | InDel | ACGTAATACTCCCCCTGAAT | CTCTATGGCCTCCATAATTG |
| In3886-5 | InDel | GGAGTAAACCAACATACCCA | GCCACAGGGCTAGATTAAG |
| In3886-6 | InDel | CATCCACAAATCTCACCTCT | ATTCAATTGGGAATTTGAGA |
| In4130-1 | InDel | TCTCTTTCAATTCTCAAAAACA | TGGTCCAAATTAAAGGTAAAA |
| In4130-2 | InDel | AGCTGTGAATCCTTCTTCAA | TACCCAATTGGTTTTGTTTC |
| In4130-3 | InDel | CACCAAAATCACTATTCACCT | GATGACAAGCCATTTGATTT |
| In4130-4 | InDel | ATACCGTTTATCTCGCACAT | TGCTTCTTCAATTACTTTGGA |
| In4130-5 | InDel | CAGATTGGGATCAATCAGTT | AGTTTTGTTTGCAGGATTGT |
| In4130-6 | InDel | GGAACAAAAACCGAACATAA | ATGGTTGTCTTGGAAAAATG |
| In4130-7 | InDel | AACTAACGCATTAGCGAAAC | CTCAGGAGCTCAATCTCATC |
| In4130-8 | InDel | ATGCTGTCTTTTCTGAAGGA | GGAAAAATCGATATAGCAGG |
| In5039-1 | InDel | CTTGGATGAAAAGCAAAAAC | GCCAAATATATCGCTAGCTC |
| In5039-2 | InDel | AATGGCGACTTCATGTTACT | GAACTAGTGCTGCTTTCGTC |
| In5039-3 | InDel | TTTATTATTCTCGTCGCACC | CGCTATGAAAACAAAGTGTG |
| In5039-4 | InDel | CACACTGAAAAATCTCCCAT | CGCTGAAGATTAAACCACAT |
| In5039-5 | InDel | CAAACTCTTTGTGCTCTGGT | CAATATGCCTGAAATTGGAT |
| In5039-6 | InDel | ATGCACTTGTCGTGCTAATA | TGATCTCCGCTAAGATGACT |
| In5039-7 | InDel | GCCGACATTAGAGATTCTTG | AACGACTTGGTGGTTGTATT |
| In5039-8 | InDel | ATCTTTGAAATCCCAATCCT | ATTGCAATGACATCAGTTTG |
| In5039-9 | InDel | GTTGCAGAAGAGAAAAATGG | ACATTCTTGCATCCTCACTC |
| In5039-10 | InDel | CCAATCTGCGGTTTACTTTA | CCTGCTACGCGTCTATTTAT |
| In5039-11 | InDel | CAACGCATCTATTAGCATCA | GTGTTCCCGATAATTAAGCA |
| In5039-12 | InDel | ACCAATTCACTTGAATCACC | ACTTTGGCATTACCGAATAA |
| In5039-13 | InDel | CATTGTTTATTGTTTCATTCCA | ATGAACGGTGTATAAATGGC |
| In5039-14 | InDel | ACAAAAACAAGTCTAAGGCG | TGGAAATCACACATAGTCCA |
| In5039-15 | InDel | TCGAATGAATAATAGAACCAAA | TACAATTTTCTGCCCATCTT |
| In5039-16 | InDel | GCAACGATATAAGTGATTTTTG | GTAGTAGTTGATGTGGCCGT |
| In5039-17 | InDel | CCGATTCTCTTTTAATGTGC | GGTGCCTTCGTACAAATAAG |
| In5039-18 | InDel | CCAACTGGAAAAAGTAATGG | GTCCTTGCTTACATAACGCT |
| In5039-19 | InDel | TCAGTGACATTCAAACCAAA | TCGAAGTCGGTTAAAGAAGT |
| In5039-20 | InDel | GGGAAGAAAGACAGTTAATGA | CCTTAAATTCAGATCTTTTCTG |
| In5039-21 | InDel | TCCGTCACCTCATATTTTTC | CATTGCAGTAGTCCCATAAAG |
| In5039-22 | InDel | GTAAAGCATTCTTGGTTTGG | CACGAATGCACTAGTACAAAA |
| In5039-23 | InDel | AAAGTACTCAACCACGCAGT | AGAAGTCAACCGCTACAAAT |
| In5039-24 | InDel | CAGTGATCACACAAAACAGG | CGTGTCACATAAACAAAAGC |
| In5039-25 | InDel | TTGTTGGCATAGTTTCCTTT | TACAACCCATGTCACACAAC |
| In5039-26 | InDel | TTTGCTGATGACTTCCTCTT | CAGAACAAAAGGTATTCGCT |
| In5039-27 | InDel | TTCAAAGAAACGCGAGATAC | TCTGGCTGGTTTTCTTACAT |
| In5039-28 | InDel | AAATTTGTGTAGGATGGTGC | TTATATCTTTCCGCCACACT |
| In5039-29 | InDel | AAAAATACAACAGGGGGTTT | CAGATCCTTGATCTTCTTCG |
| In5039-30 | InDel | ACACCCACACTAAAAATTGG | ATGTTGGAGTTTGTCAGGTC |
| In5039-31 | InDel | TGGTGATAGCTTTCTCACTGT | ATGTTGCTGGGTATCATAGG |
| In5039-32 | InDel | ACATATGAGTCCATGACCAAG | ATGTTTAATCACCACTTCCG |
| In5039-33 | InDel | ATTTGGTGAGATCATCATGG | TAACTTCACACGGACTTCCT |
| In5039-35 | InDel | AATAGCTGAAGACGCTTTTG | TAAAAGCATTTTTGGAGGAA |
| In5039-36 | InDel | AAAACTAACATTGTCTTTGGAA | GGAATCTAAGCCACTGTTTC |
| In5039-37 | InDel | GTGAGCATGATAAGTGGTGA | GGTTTGCAAATGATGACAC |
| In5039-38 | InDel | AGCAAATGTAGCAAACCATT | GGAACTCTTAGAAGCGCATA |
| In5039-39 | InDel | GCCTGATCTGGTCATTTTTA | AAGCCACTTGATTTGTTTTC |
| In5045-1 | InDel | GGCACCACAAAGAAAAATAA | CCTTGTCCTACAAGAGGACTT |
| In5045-2 | InDel | CGTAATTAGATTGTACTCGACG | TAATTGGGTCCAAGGAAATA |
| In5045-3 | InDel | GAAGACCTCTCCACATACCC | CCGTGTTAGTTCCATAGTCG |
| In5045-4 | InDel | GAAGTCCGTGATCTAATGCT | TCACTACTCACTAGCCCGTT |
| In5045-5 | InDel | TCAGCAAGAAGAAGAAGAGC | TTGTTGGAATTTTCCTCATC |
| In5045-6 | InDel | GGAGAGAGGTTGTTGACTGT | AAAATTATGGCTTGGTTGAA |
| In5045-7 | InDel | AATATTGCAGTGTCATTCCC | CGATTGGTTTCACTCAAAAT |
| In5045-8 | InDel | CGACGTTAAACTAAAATGCAC | TAGCTAGCTTCCCATCCATA |
| In5045-9 | InDel | AAACAAAATTTAAGGGGTCA | GAAAAAGATGATGAAATGCC |
| In5045-10 | InDel | TAGAGGCAGTGAGAAAAACC | ATATACCTTACTCCTCCGCC |
| In5076-1 | InDel | TTGGAGTATTGGGTATCTCG | GCTTCCTACCAGAACTAGCA |
| In5076-2 | InDel | GGATTGAGAGAGACTGGTCA | ATCATTACACCCCAAAACAC |
| In5076-3 | InDel | GAAAACGGCCATAGTAACAG | TTTTCCCACCTTTAGTGTGT |
| In5076-4 | InDel | ATTCATCCATCCATTCAAAA | TGCCAATAACACTTCCCTAT |
| In5076-5 | InDel | CTTTTCCATACTTTACACATTT | AAACTCATGCTCTCCATAAT |
| In5076-6 | InDel | CGTCTCCATTTTCTTCTCTG | ATTCGGAGCTGATTGTGTAG |
| In5076-7 | InDel | ATCCGTTACTCAAATCATGG | TTGTCTGGGAGCAAAATAAT |
| In5076-8 | InDel | TGTGTTTCTATTGCTTGATTG | CAAAGCTTATAGCCCAATTT |
| In5076-9 | InDel | CACCTGAAGCCTCAAATAAG | GCTTGTGGAAATAAAAGCTC |
| In5076-10 | InDel | CCTGGTCTACAAACACCTCT | GTGCCTTAATTTCAAACCAG |
| In5076-11 | InDel | TAGGAAGTTGCACAAATGG | AATGAATCCAAGAACCACAT |
| In5076-12 | InDel | GATTGACAAAACGACCTTAGTT | ACTCAAAATTCTTTGTGTTCG |
| In5076-13 | InDel | TTCCTCCTATGAACATCCAC | GAAAGAAACATTCCACTTGC |
| In5076-14 | InDel | GCATAAACATGGGTGTAAGG | ACTGAGCTACAAGCCATTTG |
| In5076-15 | InDel | AGCACATGCACCACATAATA | TGAGGTTATGGGCTAGATTG |
| In5091-1 | InDel | GATTTGGTCCAAGGTGATTA | CCAAGATACGTAGTTTCCCA |
| In5091-2 | InDel | TTACGAGGGGAAATCAATTA | GAAATTGAAATTGCTCGTCT |
| In5091-3 | InDel | GACTTCCCTTTTTACGGAAT | GCTATCCACACACCATTTTT |
| In5091-4 | InDel | ATGCGTACACTTGGTTTGA | TCATCCTCTTGTTAAAACACC |
| In5091-5 | InDel | CTTACCAGCAACCTATTCCA | TTGTCATGTACATTCATCTGTG |
| In5091-6 | InDel | TTTTTAGACGTGTTACCTGGA | CCAGAGAAACAAACCAACAT |
| In5091-7 | InDel | CTTATTATATCCGCCACTGC | AGCATCACTACAACGAAACC |
| In5104-1 | InDel | ACTGAAATCTTTGCCATCAG | TAGGGATTGGAACTCAAAAA |
| In5104-2 | InDel | ATCTTATGAAACGACCACCA | GTCTTATCCAACCATGAATGA |
| In5104-3 | InDel | TTCAAGCATTTCTCATAGCA | ATTTTTGGTCCTACGTACCC |
| In5104-4 | InDel | CTTACCAACGCAACCTACTC | CTGCTTGAGTTATTTTTGGG |
| In5104-5 | InDel | GCAAAAATTTATTTGTTTAGGG | CTAAGATTCATCACCGATGC |
| In5104-6 | InDel | TACATTGAAGGTGAAATCCC | ACAGACAGAAATCCTGGAAC |
| In5104-7 | InDel | CACCAAATCAATTGGAAACT | TGTTGAGGAACTTTGTGAAA |
| In5104-8 | InDel | CAACACTTCTAGCAACCCTT | GGGAAGAAAGTGAAGAAGGT |
| In5104-9 | InDel | GCCAAATTAAAATGTGGGTA | CACCATTTTATTAAGCCACC |
| In5104-10 | InDel | CTCTACGGTTTGAAGCAATC | AATGAACACACAATGGCATA |
| In5104-11 | InDel | TATTTTGGGACTTCAGCAAT | GTGTGTGTAGACGGAAAGGT |
| In5104-12 | InDel | TGATGTAATGCAAGTATGCAA | CATCATGAGACAACCATTGA |
| In5104-13 | InDel | TCATCGAGGTTTGAGATTTT | ACATTTCCATTTCATATCCG |
| In5104-14 | InDel | ACTTCAGGTCAAGGCATAAA | CGTTGACACGAATCTCACTA |
| In5104-15 | InDel | GGCGTCAATATTTGCTACTC | TGACATGCACAGCTTTAGTC |
| In5104-16 | InDel | TTTATGAACTCTCTGTCACCTT | TTCTCTTCATTTTTGGGTTT |
| In5126-1 | InDel | GAGCATGTTGTTCATGTTTG | GGGTTTTTCATTCGTCAATA |
| In5126-2 | InDel | GCTATTTTGAAATGCCGTAT | TCCTTCCCAAGTTATTTGAA |
| In5126-3 | InDel | CAAACATGCCAACTCTTAGG | ACAACATTTGACAAGGGAAG |
| In5126-4 | InDel | GCTTCCTGAGAGAAGATTGA | ACGACAACATCAAACAGTGA |
| In5126-5 | InDel | GGAATCAAAGAGGAATGTGA | AGACAAATCGTATCTCACGG |
| In5126-6 | InDel | GGTTCGACATTACACCAAAC | CGTTTGTTGTACCGGATTAT |
| In5126-7 | InDel | TTGACCTTCCTATCATGTCC | GAGATGGTGATGTGATGTGA |
| In5126-8 | InDel | ATACGAACGAACCTGAAGAA | TATAGCACTCACACCCTCCT |
| In5126-9 | InDel | GGATGTGAACGGTAATTGTT | CTTTTCCAATAAAAGCTGGA |
| In5126-10 | InDel | GTTCTAAAAGACGTTAGGCG | ACTTAAATCCGTCTAATCCG |
| In5126-11 | InDel | GAATAATTTGACTTCGCTGG | TTTAGTGTCAATGTGGCTTG |
| In5126-12 | InDel | GGGGATGAAGGGTATAAAAC | ATCAGGCAATTTCACTTTGT |
| In5126-13 | InDel | TTCCGCAGAGATTACTGTTT | TAAAGTTGTTCCTGCCTGTT |
| In5130-1 | InDel | AAAATGGTCACAAAGACGAT | ATCAATTGCTTGATAATCGG |
| In5130-2 | InDel | TGTTGTTTGTGGTTGTTTTC | GAAGAAGCGTACCATTTCAC |
| In5130-3 | InDel | CTAATTCTTCCAAGTCCACG | GCACCTGATTCTCTTGAAAC |
| In5130-4 | InDel | TTATGAGCAGAAAAGCTTCC | AGCAAACAAAGCTAAACCAG |
| In5130-5 | InDel | AAATAGCACACAACCCTGTAA | ACATAGGACATGGATTCTGG |
| In5130-9 | InDel | TAAGCCAAAACCATCAAGTT | TTGAATGTATAATGGACCAATG |
| In5130-11 | InDel | AATTCTTTTAAGCAAATCGTT | CAAAAGAGGGCTTCAGTAAT |
| In5141-1 | InDel | CAAAACAGGAAGACACATGA | AACCATGCGTTGACTAAAAT |
| In5141-2 | InDel | GCAGAAATTCAATAACGAGAA | TGTACGATAAACGGACACAA |
| In5141-3 | InDel | TTGAAGAGGTTGGTTGACTC | GTTTTGTAGTCAATCCCCAA |
| In5141-4 | InDel | ATCTCCAAGCGATGATAATG | AACCTGAGAGAACCCTTTGT |
| In5141-5 | InDel | TACTACCCCTAATGGCTTGA | AACGCAATATTAAACCGAAA |
| In5141-6 | InDel | ATTTATAGGCTCCCCAAAAC | CCATTGGCTTTTCTAGTTTG |
| In5141-7 | InDel | GCATGATTAGGAATTTTTCG | TCCCAACCTAGCCAATAATA |
| In5141-8 | InDel | ATCTTACAATCCACCTCCCT | GAGAATATCGTGCTACGGTG |
| In5141-9 | InDel | AACCCTGTAAACATGCAAAC | CAATGTGGGACTCTTTTACC |
| In5141-10 | InDel | TGAACGTTTGTGATGCTTAC | AAGCTTGTCCCGACTTTAG |
| In5461-1 | InDel | TTTTTATGCGAATTTTGATT | AGATTCTTCACCACCAAGTT |
| In5461-2 | InDel | GAGTTACTGGGGGAAAAATC | CAAGCTCAAACTGGCTTTAT |
| In5461-3 | InDel | GTTTTTCCTCAAGGGTTCTT | ACTAGAGAACCGAATCATCG |
| In5461-4 | InDel | ACCTAGATCTGCCCTCATTT | GGAAAGAAGAGCTAAAAGGG |
| In5461-5 | InDel | ATATAATCCGAGCAACACCA | ATCGATGTTTACCACCTCTG |
| In5461-6 | InDel | TCGTTGGTTCCCTAATCTAA | TACAGTTAGCAATGGTGCAA |
| In5660-1 | InDel | ATAACGCTTGTCCATGAAAC | GGGAAGAATAAGGATATGGG |
| In5660-2 | InDel | AAGGAATGTAGCCATTGAAA | GTCTCGGTTCTGGTATTGAA |
| In6478-1 | InDel | AACTTGTATTCGACAGGACG | CGATTAGTTTAAAATTGCGG |
| In6478-2 | InDel | GAATATTTGGCATCATTTCC | TTTTTCATTTTCTTTTTGGG |
| In6478-3 | InDel | ACGTGCGTATACAACAAACA | CGATTCAAATCAGCCTATTC |
| I7041-1 | InDel | AAGATTAAGGCCTATCCACC | GAACATAGTCCAGCCCAATA |
| I7041-2 | InDel | GAAAGTGCCAGGTAGTGAAG | GAAAATCCCATCAAAATCAA |
| I7041-3 | InDel | CATCCTGTGGCAGAGATATT | TTAGTAGAACAGCTCCTCCG |
| I7041-4 | InDel | AGGTTGTGAGTTTTGCATTC | AAGGAACATGAAGACTGACG |
| I7478-1 | InDel | GACACAATCTCGAACCAAAT | GCAGCTCGTTTTATATGTCC |
| I7478-2 | InDel | CAGAATATCCTGCAAATAAAAA | ATCTGGGCAAACTCTGAATA |
| I7581-1 | InDel | TCTCCCCTGAACAAATACAC | TCATTGACTCATGGATGCTA |
| I7581-2 | InDel | TCACATCATAATCTCTTGCG | TATAAGGGCATCTTCAAAGG |
| I7715-1 | InDel | ATTTTCCCACTGAGTGAATG | TAAGGGTAAACCTGAGTTGG |
| I7715-2 | InDel | TTGTTTCTTTTGCATCTGTG | TCGGGGTTAAGTGAATCTC |
| I7715-3 | InDel | TCCTTCGCTTTAATTTTGTC | TGCTCCTTGTTTCACTAAAAA |
| I7715-4 | InDel | TGTGCAAATTTCTGAATACG | TTCATTAGAACCTGCCAAAT |
| I7715-5 | InDel | CAGTGAAAAATTCTACGAAAG | GAGAGAGAGAGAGAGAGAGAGA |
| ZFRI130-16 | InDel | CACTTTCGAAGCCAACAAAT | GGGCAAACTGGGAAACTG |
| ZFRI130-1 | SSR | ATCACTTTGCTTCCTAATGC | CCTCAACGATTCAGACTCA |
| ZFRI130-2 | SSR | AATCGCAGCCAGAAGAAG | GTATCGCAGTATCGCACT |
| ZFRI130-3 | SSR | GGAGGACGAGACCTTCAA | CCTTCAATTCACTCAACCTAC |
| ZFRI130-4 | SSR | GCTCCTCTGCAATGTTGA | CCGCTTCCATCTGATATAATC |
| ZFRI130-5 | SSR | AATAAGCACCACAGTTAGC | TCTTCTCCATACGCAACAA |
| ZFRI130-6 | SSR | TCCAAGGTTAGTAAGGTTCA | GGTTGTCAGTGAGATATGC |
| ZFRI130-7 | SSR | GTGTGAATAGCACCACTTAT | TGGGCTACTTTCTTGTAAAC |
| ZFRI130-8 | SSR | TGCTCCACCTTCTGATAGA | CTCCGAAATGAATAATTGCC |
| ZFRI130-9 | SSR | ACCAGCGTAGTTTGATAAGA | AAGGTCGTCAAGTGAGAAG |
| ZFRI130-10 | SSR | TCCTTGATAGTTCAGTCGTT | ATAGATAGGTCTACTCCTCAAC |
| ZFRI130-11 | SSR | TGCTCACCATGTTTCAGAA | TTAAGTATGCGTGTGCGT |
| ZFRI130-12 | SSR | ATCGGAGTGTGTGGATATAG | AAATGGGCAGAGAACTCAA |
| ZFRI130-13 | SSR | CGGAGTGTGTGGATATAGAG | GGCTGCGTTCAATCAATC |
| ZFRI130-14 | SSR | GAAGAGCAAGATACACTAGAGA | TAGTTAGAGGTGAGCTGTCT |
| ZFRI130-15 | SSR | TATTGAAACCTCACATACCG | GAACTGGCAGAATCTAACG |
| ZFRI130-17 | SSR | GGACATTACAGCAGCATTAC | GTATTGTTCTCTGGTTATGGAC |
| ZFRI130-18 | SSR | TGATGAATGTACCATGTTTATAC | GTCGTAATGTATTTCTATGTATG |
| ZFRIt118 | SSR | CAGGTGAGTGGGCTTTTGAT | CTCCGCAGCGTCATACAATA |
| ZFRIt206 | SSR | TTCATGCATATGGACCCTCA | TTTGGTGGAGGAGTGGAGAG |
| ZFRIt239 | SSR | TTTGCGGCCTTCAAACTAAT | TCGTTGAGACGTCGATGAAG |
| ZFRIt240 | SSR | CACTCACTGTGCTGGTAGGG | AGCGCTTCAAAGTTTCCAAC |
| ZFRIt243 | SSR | TCAATGTGAAGTGGGTTTCG | ACCGCAACTATCCCTCTCCT |
| ZFRIt380 | SSR | CGATGCGTCATGAGTTTTGA | GCAGCACCTACAGTGCCTTT |
| ZFRIt418 | SSR | CCCCATTTTTGTGTGTCCTC | AGTGTGCATTTGTCATGTTGC |
| ZFRIt422 | SSR | CCGGTGTCTGTTGTGAATGT | CGAGAGCAAAACTCATCGTG |
| ZFRIt433 | SSR | TGGTCATGTGAGAGGAGGTG | AAATCACACCCAACACGACA |
| ZFRIt460 | SSR | GTGCCACGTAGGCTTTGTAA | GGACTTGGGCTTGCTACATC |
| ZFRIt477 | SSR | CGAGAGCGCCTAAGAGAAGA | CGGAGACTCGCTGACTCACT |
| ZFRIt489 | SSR | AACGAAGTCTTAGCTGAAGGTGA | CAACTCAACTCCCCCGTTTA |
| ZFRIt510 | SSR | TTAATTTCCTTCGGCCTTCA | GGCGTCTTGCAAACATAGGA |
| ZFRIt514 | SSR | GCTTTTGGCACTTTGGATTC | CGGTTGCATCAGACTTTCAG |
| ZFRIt535 | SSR | GAGAGAGAGAGGGGAAACGAG | GGCTGCGTTCAATCAATCTC |
| ZFRIt536 | SSR | GTTGGCCATAACCATCACCT | CTTCGAGCTCTTCTGCTGCT |
| ZFRIt537 | SSR | TTGCTTGAAGAGCAAGATACACT | TGCAATCTCAAAAGTCACAGC |
| ZFRIt538 | SSR | CCCCAACACTGGACCTACAT | TTGCGAAGGGAAAGGTACAG |
| ZFRIt539 | SSR | TTGAAGAATGTACCCATGTTTTG | CCTTGTTGGTTTTCAATTGTTG |
| ZFRIt540 | SSR | TGTCAACCAAAAGAGGGTGA | GGGGAGGTTTTTGTTTTCG |
| ZFRIt541 | SSR | AAAAACCGAAAACAAAAACCTC | CGGAGCCCTGTGCTACTAAT |
| ZFRIt542 | SSR | CCGATCTTTTATCAAGCACCA | TGCATATGGGGTGTGATTTTT |
| ZFRIt543 | SSR | ACTAATCACCCCCACACACA | TCCCACGCCTTTAAGAATGT |
| ZFRIt544 | SSR | AACAAGAAAGCAAAAAGCCATT | GCTGGGAAGATGATTTGAGC |
| ZFRIt545 | SSR | GCGAGCATGCAGTAAGTGAG | GGAGCCAGTTCGTCACAAAT |
| ZFRIt546 | SSR | TTTGTTCGTAAGAATCGACTTGA | GGGCATGTGCTTCAAATTCT |
| ZFRIt547 | SSR | TGGAAGTGCCAAGTTAAACTCTC | TGGTCAAAAAGAGGCTGAAA |
| ZFRIt548 | SSR | TACCAGAGATCTGGGCAACC | TGGACCAGGGCAATAGTTCT |
| ZFRIt549 | SSR | TTTTTCTCCCCATTGGGTTT | GATTCGCTTCCCACTAACCA |
| ZFRIt550 | SSR | TCAAGAACGAAGCGAGATCC | CTTCCTCCCATTCAATGTCC |
| ZFRIt551 | SSR | AATCCAAAGTGCAACCAACC | TGAAACTTCCTACGGGTGATG |
| ZFRIt552 | SSR | TTTTGCAGCTCGTTGATTTG | CCTGCCAAAAACCAACTTTC |
| ZFRIt553 | SSR | TTCTTCTGGCCACTCCTGTC | TCCTGATTTCCCTACCGTTG |
| ZFRIt554 | SSR | TTTTGACCAATTAGAGCCACA | GATCCAAGGATCTGGTGTGC |
| ZFRIt555 | SSR | AGTAAGCATTTCGGGCAAGA | ACAACTTCCCCACAATCTCG |
| ZFRIt556 | SSR | CCCCATTTCTTTTGGGTTTT | TCATTAGACGTGGGTGGTGA |
| ZFRIt557 | SSR | CATCACCCGTAGGAAGTTTCA | TTTGCCCTCCTTTTGTCAAT |
| ZFRIt558 | SSR | CAACCATTATTCGCCACAGA | TTCACTTGACCCACCACATC |
